# Rare Presentation of Multiple Non-syndromic Supernumerary Premolars Affecting All Quadrants

**DOI:** 10.7759/cureus.94857

**Published:** 2025-10-18

**Authors:** Namrata Jain, Debapriya Pradhan, Saurabh Tiwari, Ankit Dhimole, Nikita Saini, Aishwarya Jethi, Avni Shrivastava, Delphina Michael Kapoor

**Affiliations:** 1 Department of Pedodontics and Preventive Dentistry, Hitkarini Dental College and Hospital, Jabalpur, IND; 2 Department of Oral Medicine and Radiology, Hitkarini Dental College and Hospital, Jabalpur, IND

**Keywords:** clinical pediatric dentistry, non syndromic, orthopantomograph (opg), premolars, supernumerary teeth

## Abstract

Supernumerary teeth are developmental anomalies that may occur anywhere in the dental arch, most frequently in the maxilla. While single or few supernumeraries are relatively common, the presence of multiple supernumerary premolars in all quadrants is an extremely rare phenomenon, particularly in non-syndromic patients. This report describes a 12-year-old male presenting with dental caries, in whom routine radiographic examination revealed multiple supernumerary premolars distributed across all quadrants. Relevant literature is also discussed to highlight the rarity, clinical implications, and management considerations of such cases.

## Introduction

Supernumerary teeth, often termed “extra teeth,” represent a developmental dental anomaly of uncertain etiology. Various theories, including phylogenetic, tooth germ dichotomy, hyperactivity of the dental lamina, and multifactorial genetic-environmental influences, have been proposed to explain their origin [1,2]. These teeth may occur in both jaws, though the maxilla is the most common site [3]. Reported prevalence ranges from 0.1% to 3.8%, with the permanent dentition more frequently affected [4]. Supernumerary premolars constitute approximately 8-9.1% of cases, predominantly in the mandible [5]. Morphologically, they often resemble normal premolars and are classified as “post-permanent” teeth, arising later due to continued dental lamina activity [6].

Supernumerary teeth may present in various morphologies - conical, tuberculate, molariform, or odontomas - and can erupt as mesiodens, paramolars, distomolars, or parapremolars. Clinically, they are associated with delayed eruption, malalignment, cyst formation, root resorption, or complications during orthodontic and implant therapy [3].

Gardiner [7] described three mechanisms for supernumerary premolar development: Type A (early lamina proliferation producing pre-deciduous teeth), Type B (formation of an additional follicle before the permanent tooth), and Type C (post-permanent type, developing after the normal follicle has formed). Among these, Type C supernumeraries typically erupt 7-10 years later than their counterparts, often as “delayed surprises” [7]. This report highlights a rare case of non-syndromic multiple supernumerary premolars identified incidentally in all four quadrants of a young patient.

## Case presentation

A 12-year-old male patient reported to the Department of Pediatric and Preventive Dentistry with a primary complaint of intermittent pain in the lower left posterior region for the past two days. The patient was of average build and appeared cooperative during clinical evaluation. His medical history was unremarkable, with no known systemic illnesses, hospitalizations, or long-term medication use. Family history did not reveal any similar dental anomalies, and there was no reported history of hereditary conditions or syndromic features. On extraoral examination, no abnormalities such as facial asymmetry, swelling, or cutaneous stigmata suggestive of syndromic association were observed. Temporomandibular joint movements were within normal limits.

Intraoral examination revealed a mixed dentition stage. The oral hygiene status was fair with localized plaque deposits. Carious lesions were detected: a Class II carious lesion involving the mesioproximal surface of tooth 75 and a Class II carious lesion on the distoproximal surface of tooth 84. Adjacent gingiva appeared healthy, with no signs of swelling, sinus tract, or periodontal pocketing. To further evaluate the extent of carious involvement, radiovisiography (RVG) was performed in the third and fourth quadrants. Incidentally, unerupted supernumerary teeth were noted in both quadrants. Considering this unexpected finding, an orthopantomograph (OPG) was advised for a complete evaluation.

**Figure 1:**
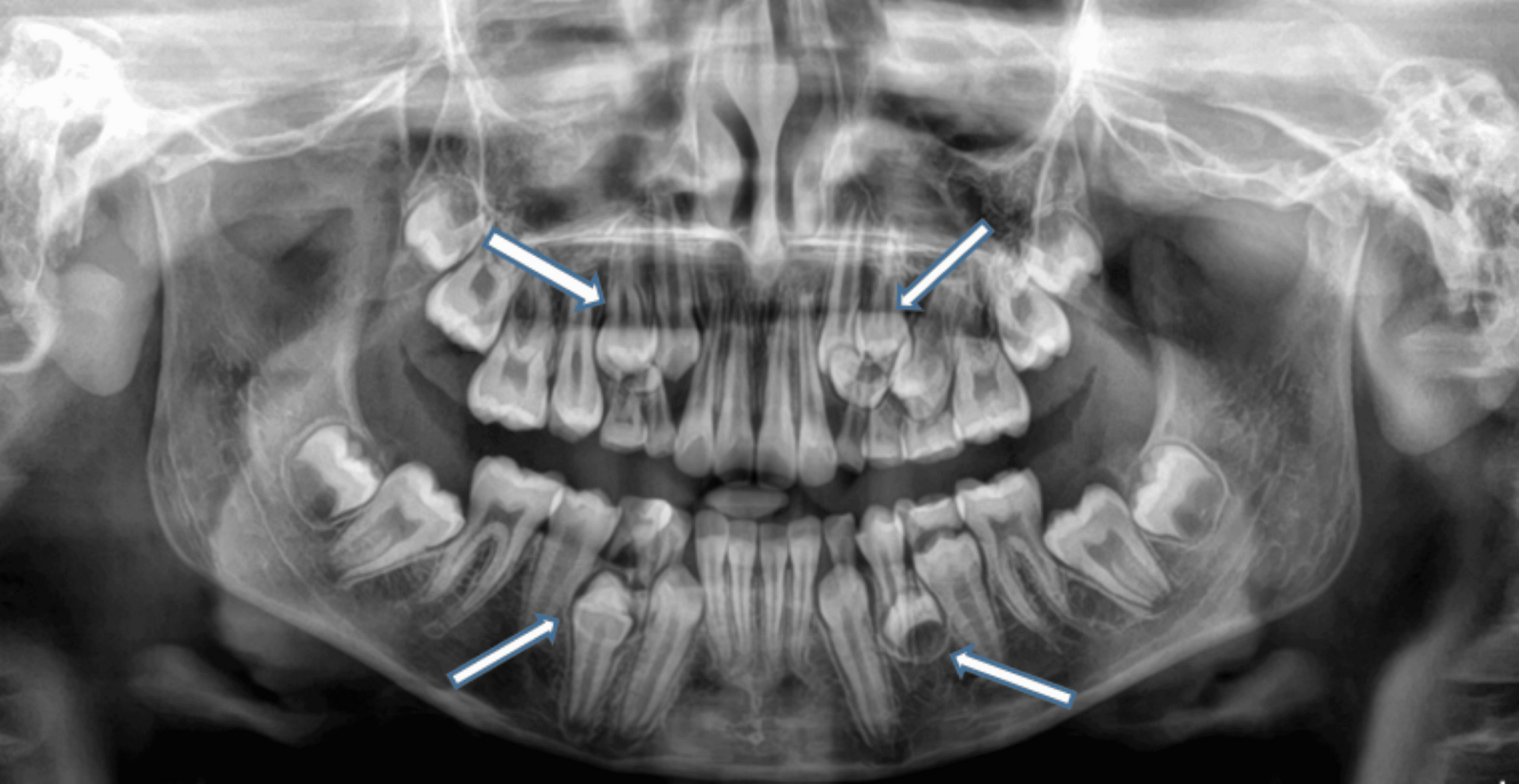
Orthopantomograph showing supernumerary premolars in all four quadrants. The supernumerary teeth are superimposed on the first premolars in the first, second, and fourth quadrants, while a developing supernumerary bud is evident in the third quadrant.

The panoramic radiograph confirmed the presence of multiple supernumerary premolars distributed across all four quadrants (Figure 1).

Specifically, supernumerary teeth were superimposed on the first premolars in the maxillary right, maxillary left, and mandibular right quadrants, while a developing supernumerary tooth bud was evident in the mandibular left quadrant. No blood or biochemical investigations were deemed necessary as the patient was otherwise healthy, and a systemic or syndromic association was clinically ruled out. The provisional diagnosis was multiple non-syndromic supernumerary premolars in all quadrants, with concurrent caries in teeth 75 and 84.

The treatment plan was formulated in two phases. For immediate management, the carious lesions in teeth 75 and 84 were excavated and restored with composite resin to relieve pain and prevent further progression of decay. For long-term management, since the unerupted supernumerary premolars were asymptomatic and not interfering with eruption or occlusion, a conservative approach of periodic monitoring was advised. The patient was scheduled for six-monthly clinical and radiographic follow-ups, and his parents were counseled regarding the nature of the anomaly, potential future complications such as eruption disturbances, crowding, or cystic changes, and the possible need for surgical intervention should these arise.

## Discussion

Supernumerary teeth are relatively common, yet the occurrence of multiple supernumeraries is rare, with a prevalence of five or more teeth reported in less than 1% of cases [8]. In the Indian population, the frequency of impacted supernumerary teeth is estimated to range from 0.1% to 1.2%. Such multiple occurrences are often associated with syndromes, including cleidocranial dysplasia, Gardner’s syndrome, orodigitofacial dysostosis, Down syndrome, Crouzon’s disease, and Hallermann-Streiff syndrome. Nevertheless, non-syndromic presentations, such as in the present case, have also been documented [9].

Supernumerary premolars account for approximately 8-9.1% of all supernumerary teeth and are predominantly found in the mandible [10]. They are usually morphologically similar to normal premolars but tend to erupt later than their permanent counterparts. While premolar calcification typically begins between 1.5 and 2.5 years of age and becomes radiographically visible by 3-4 years, supernumerary premolars may not erupt until seven to eleven years later [3]. In the current case, multiple unerupted supernumerary premolars were evident in all quadrants in a 12-year-old child, highlighting the importance of radiographic evaluation.

Arandi [11] reported that 57-90% of supernumerary premolars occur in the mandible, and similar trends have been described in Indian literature. Mehra and Rekhi [8] reported bilateral erupted premolars in an 18-year-old male, while Tanwar et al. [9] presented five cases of non-syndromic multiple supernumerary premolars. Kaur et al. [12] documented a unilateral mandibular supernumerary premolar with a dilacerated root, and Sarkaria and Ogadako [13] described a dizygotic twin with multiple supernumerary premolars, distomolars, and anterior maxillary teeth. Kallury et al. [14] also reported a case of non-syndromic supernumerary premolars, whereas Mali et al. [15] conducted a study analyzing their prevalence and characteristics in non-syndromic patients. Additional reports include a molariform supernumerary tooth by Shashikiran et al. [16], a bilateral occurrence of para-premolars in both jaws by Kothavade et al. [17], and a familial occurrence in two sisters described by Kumari et al. [18]. These reports emphasize the variability of presentation and support the rarity of the condition observed in the present case.

Management of supernumerary teeth is guided by their morphology, location, and impact on adjacent structures. They may lead to complications such as delayed eruption, displacement of permanent teeth, or caries in adjacent teeth. Treatment options include extraction, endodontic therapy, orthodontic intervention, or periodic monitoring. Extraction is generally recommended when pathology, functional disturbance, or esthetic concerns are present. In contrast, asymptomatic teeth that do not compromise occlusion or development may be retained with periodic follow-up [3,19].

Alshammari and Madfa [19] highlighted successful management through comprehensive surgical removal of maxillary supernumerary teeth followed by orthodontic traction, which achieved favorable alignment and esthetic outcomes. In the present case, a surgical plan was advised once eruption occurred, with the recommendation for regular follow-up and orthodontic assessment as needed.

## Conclusions

This case illustrates the rare occurrence of multiple non-syndromic supernumerary premolars affecting all four quadrants in a young patient, discovered incidentally during routine radiographic evaluation. The report underscores the importance of thorough clinical and radiographic assessments, even when patients present with seemingly routine complaints, as unexpected anomalies may be detected. Early identification of such teeth is crucial, since they may remain asymptomatic for years yet predispose to complications such as delayed eruption, malocclusion, crowding, or cyst formation. A balanced approach, combining immediate management of presenting dental problems with vigilant long-term monitoring of asymptomatic supernumeraries, is essential to prevent future complications. This case highlights the value of routine imaging in pediatric dentistry and reinforces the need for individualized treatment planning to ensure optimal outcomes.
